# Alcohol‐related liver disease mortality and missed opportunities in secondary care: A United Kingdom retrospective observational study

**DOI:** 10.1111/dar.13482

**Published:** 2022-05-31

**Authors:** Mohsan Subhani, Rebecca Elleray, Jane Bethea, Joanne R. Morling, Stephen D. Ryder

**Affiliations:** ^1^ Nottingham Digestive Diseases Biomedical Research Centre, School of Medicine University of Nottingham Nottingham UK; ^2^ NIHR Nottingham Biomedical Research Centre Nottingham University Hospitals NHS Trust and the University of Nottingham Nottingham UK; ^3^ Public Health England, Seaton House Nottingham UK; ^4^ Population and Lifespan Sciences, School of Medicine University of Nottingham Nottingham UK

**Keywords:** alcohol‐related liver disease, mortality, retrospective

## Abstract

**Introduction:**

Alcohol‐related liver disease (ARLD) is a preventable cause of mortality. Historical epidemiological studies on ARLD often lack a detailed linked assessment of health‐related contacts prior to death which limits understanding of opportunities for intervention. We aimed to analyse retrospective population‐based data of all adult residents of Nottinghamshire dying from ARLD to determine the factors associated with delayed diagnosis of ARLD and the potential missed opportunities for interventions.

**Methods:**

We linked the Office for National Statistics and Hospital Episode Statistics databases to identify adult (≥18 years) residents of Nottinghamshire, who died of ARLD over the 5‐year period (1 January 2012 to 31 December 2017). Death was used as the primary outcome, and logistic regression analysis was conducted to test the association between key variables and mortality due to ARLD.

**Results:**

Over 5 years, 799 ARLD deaths were identified. More than half had no diagnosis or a diagnosis of ARLD less than 6 months before death. Emergency presentation at first ARLD diagnosis and White ethnicity were significantly associated with a delay in diagnosis. Overall, the cohort had a median of five hospital admissions, four accident and emergency attendances and 16 outpatient appointments in the 5 years before death. Treatment was provided by a range of specialities, with general medicine the most common. Alcohol was associated with most admissions.

**Discussion and Conclusions:**

This study identified deficiencies in ARLD secondary care and provides us with a powerful methodology that can be used to evaluate and improve how alcohol issues are managed and where action can be best targeted.

## 1 INTRODUCTION

The alcohol related mortality rate in 2020 in the United Kingdom (UK) was the highest reported since 2001, with a 19.6% increase compared to 2019 [1]. In 2018, the World Health Organization reported that alcohol use contributed to over 3 million deaths (men 2.3 million, women 0.7 million), and 132.6 million disability‐adjusted life years globally per year [2]. Despite this global rise, significant geographical variation persists; for example between 1990–2014 alcohol consumption in most parts of Europe either remained stable or declined, however, in the UK and Finland it significantly increased [3]. Harmful alcohol intake has substantial social, economic and health consequences [4]. In the UK, an annual 1.3 million alcohol‐related hospital admissions are costing an estimated £3.5 billion per year to the National Health Service (NHS) [2, 5]. The UK has observed a 400% rise in mortality due to liver disease over the last three decades. It is now the third most common cause of premature death and the second most common cause of working life years lost in men and the fifth in women [2, 6]. It is important to highlight that alcohol interventions have been in practice in the UK for over 50 years, but the current dismal situation reflects the need for more effective measures to mitigate the alarming rise in alcohol‐related harm [7, 8].

Alcohol‐related liver disease (ARLD) is asymptomatic in the early stages and often presents late when the prognosis is poor with limited interventional impact [9]. Recent evidence from UK Biobank data has shown individuals with ARLD to be 12 times more likely to present late compared to those with viral hepatitis [9]. Once patients with alcohol misuse develop cirrhosis the prognosis becomes exceptionally poor, with mortality as high as 75% at 5 years and 91% at 15 years [10]. However, the liver has remarkable recovery potential; stopping drinking is the key factor in improving outcomes and survival for those with liver damage and remains the mainstay of treatment [11].

Early identification of alcohol misuse is therefore key and there may be numerous opportunities to identify alcohol misuse and/or diagnose ARLD earlier. Alcohol is related to over 200 different medical conditions and is a common cause of hospitalisation [2] across many different clinical specialities [2, 12]. Such attendances may represent missed opportunities for an earlier diagnosis of liver disease in this ‘at‐risk’ population and for evidenced‐based interventions for alcohol misuse to reduce the risk of presentation with complications of cirrhosis [13]. In 2019, 7.4% of all hospital admissions in England were related to alcohol [6, 14, 15]. In 2020–2021, 18% of all patients admitted to Nottingham University Hospitals NHS Trust were screened positive for alcohol use disorder and of them, 4% were alcohol dependent [12]. Early identification of ARLD followed by intervention is the most successful way to reduce alcohol‐related harm, with the availability of multiple non‐invasive tests to reliably check for liver fibrosis, it is important we make every contact count [10, 13]. Studies have demonstrated that if current treatment figures for alcohol dependence can be increased from 8% to 40%, it will reduce alcohol‐related mortality in men by 13% and women by 9% [16].

There is limited research describing where and how patients with underlying ARLD interacted with health‐care professionals. Moreover, little is known about specific factors associated with delayed diagnosis in ARLD [9, 10]. Historical epidemiological studies on ARLD often lack a detailed linked assessment of prior health‐related contacts and associated demographic and clinical factors, limiting understanding of opportunities for intervention.

We aimed to analyse retrospective population‐based 5 years linked Hospital Episode Statistics (HES) and Office for National Statistics (ONS) data of all adult residents of Nottinghamshire dying from ARLD to determine the factors associated with delayed diagnosis of ARLD and potential missed opportunities for the interventions.

## 2 METHODS

### 2.1 Data sources

HES contain statutory patient information for a range of events, including all hospital admissions, out‐patient department (OPD) appointments and accident and emergency (A&E) attendances for NHS hospitals in England. The HES database contains a variety of information on individual patients for each event, such as primary diagnosis (based on the International Classification of Diseases, 10th edition; ICD‐10), the speciality of admission, demographics and mode of admission (emergency/elective). ONS provides death registry data which is linked by NHS Digital to the HES database.

### 2.2 Study setting and population

Nottinghamshire is a county in the East Midland region of England with a population of 817,900 (mid‐2017). This includes Nottingham which is the biggest city in Nottinghamshire with a mid‐2017 population of 329,200 [17].

The cohort was defined as adult (≥18 year) residents of Nottinghamshire (including Nottingham City) as determined by postcode, with death registration between 1 January 2012 to 31 December 2017, and ARLD in any cause of death field.

ARLD was defined as any of the following ICD‐10 codes: K70.0—alcoholic fatty liver, K70.1—alcoholic hepatitis, K70.2—alcoholic fibrosis and sclerosis of the liver, K70.3—alcoholic cirrhosis of the liver, K70.4—alcoholic hepatic failure, K70.9—alcoholic liver disease, unspecified. Conditions were defined as wholly alcohol‐attributable where alcohol was the sole cause and their alcohol‐attributable fraction was 1.0 (100%), as per Public Health England (PHE, 2014 and 2020) guidance [18]. The detail on alcohol‐specific conditions included in the study is provided in Table S1 (Supporting Information).

Through the ONS‐HES linkage for all individuals meeting the inclusion criteria, we obtained the data on the following variables: date of death, age at death, sex, ethnicity, lower‐layer Super Output Areas (LSOA) of residence at the time of death, type of ARLD (ICD‐10), number of inpatient admissions, A&E attendances, OPD attendances and speciality of care.

The LSOAs are produced by the ONS to describe statistics of a small area with an average of approximately 1500 residents or 650 households. As per the 2011 census, there are 32,844 LSOAs in England which were used to determine indices of multiple deprivations 2015. More detail can be found on the Ministry of Housing, Communities and Local Government webpage [19].

Deprivation was assigned by using the index of multiple deprivation 2015 quintile as provided by PHE based on the LSOAs of residence at the time of death. The index of multiple deprivation decile combines information from seven domains and produces an overall measure of deprivation. The index of multiple deprivation ranks the scores to produce quintiles with 1 equal to most deprived 20% and 5 equal to least deprived 20% of neighbourhoods nationally.

The date of incident ARLD diagnosis was defined as the first date that a K70 code (as above) appeared on any HES record for inpatient admission, A&E attendance or OPD attendance. If the patient was first diagnosed with ARLD during an inpatient admission the mode of admission (emergency or elective) was noted.

### 2.3 Data source

Access to the linked HES‐ONS Mortality dataset was gained as part of the Internal PHE Data Access Agreement as per the conditions of use in the Health and Social Care Information Centre guidance [20, 21] which were in place at the time of the study.

The linkage was carried out by a dedicated NHS Digital team. It is based on matching the date of birth, NHS number, sex and home address information between the two datasets. The linkage process results in assigning a unique HES patient identifier to the ONS death record. This unique identifier then is used to link to HES OPD, A&E and inpatient data.

### 2.4 Missed opportunities for intervention

Potential opportunities for intervention were defined as any inpatient admission, A&E attendance or OPD attendance in the 5 years before death. For each event in that period, we extracted the admission/attendance date, the associated diagnoses (primary and secondary using ICD‐10) and the speciality of attendance (not for A&E attendance).

In addition to the overall analysis, participants were divided into an “early diagnosis” group where cirrhosis was diagnosed more than 6 months before death, and a ‘late‐diagnosis’ group where the diagnosis was made less than 6 months before death. This was undertaken as the opportunity for intervention by reducing alcohol intake was thought limited in those already in the last 6 months of life. By contrast, those surviving more than 6 months from an initial diagnosis of ARLD would have a significant chance of clinical benefit if alcohol cessation interventions were undertaken.

### 2.5 Analysis

The statistical analysis was undertaken using Stata statistical package (software version 13.1; Stata, College Station, TX, USA; Computing Resource Centre, Santa Monica, CA, USA and R version 3.5.2; 20 December 2020). The normally distributed variables were expressed as mean ± SD, non‐normally distributed variables as median with interquartile range and categorical variables as frequency. Descriptive analysis was used to describe the distribution of key variables among the study population. Death was used as the primary outcome, and a univariate and multivariate logistic regression analysis was carried out to test the association between key variables (age, sex, area of residence, deprivation quintile, ethnicity and mode of admission) and delay in diagnosis of ARLD. The variables were mutually adjusted for each other (Table S2, Supporting Information).

### 2.6 Ethical approvals

The ethical approval for access to HES and ONS linked data was gained as part of the Internal PHE data access agreement as per the conditions of use in the Health and Social Care Information Centre guidance [11, 20, 21]. Nottingham University Hospitals NHS Trust provided local approval for the project as a service evaluation. Additional approval from the Division of Epidemiology and Public Health at the University of Nottingham was obtained on 15 March 2019 allowing this work to be undertaken as part of a Master of Public health dissertation project by RE.

## 3 RESULTS

### 3.1 Characteristics of the cohort

**FIGURE 1:**
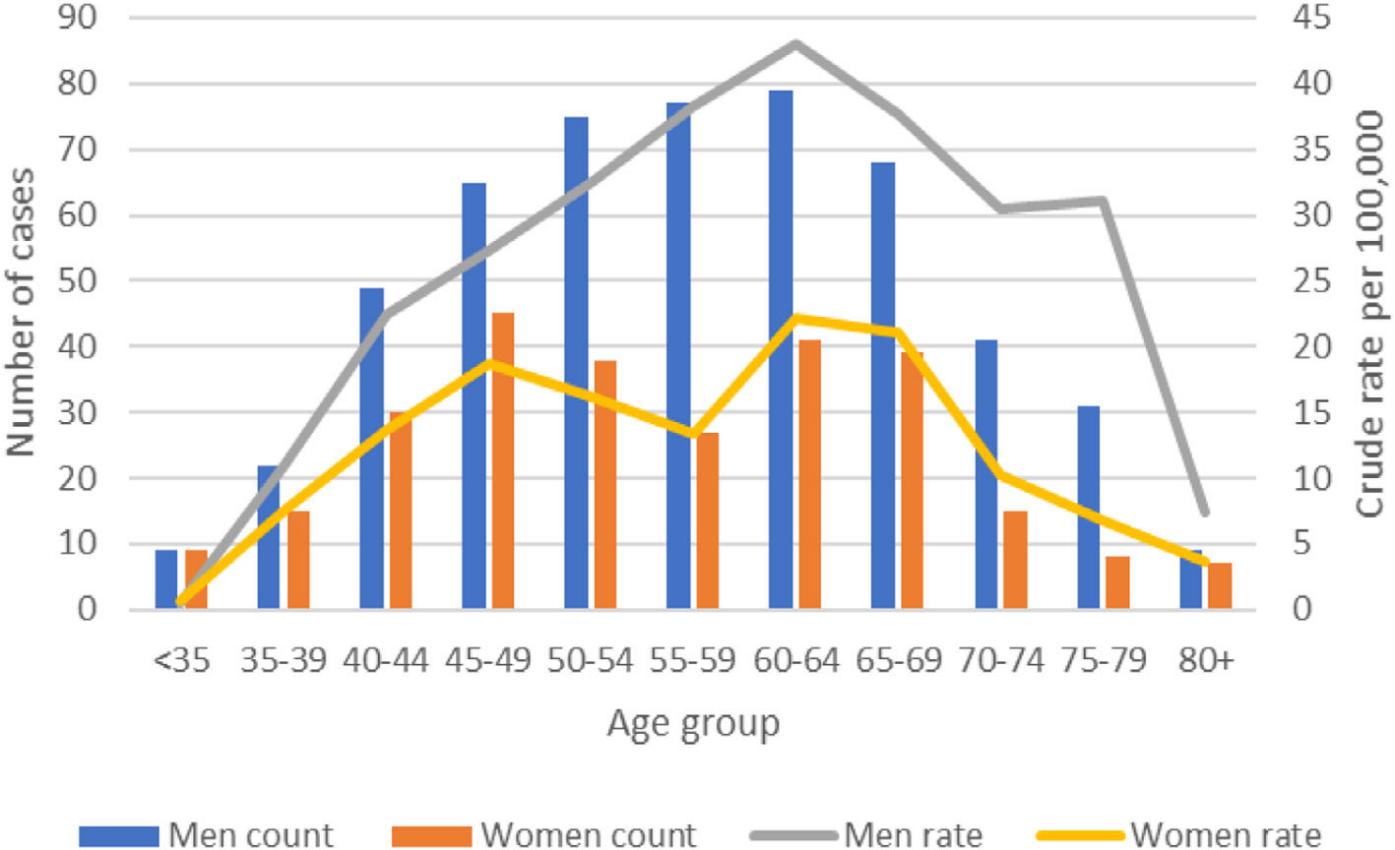
Number of cases and crude rates by age group for men and women, Nottinghamshire‐wide, 2012–2017

Seven hundred and ninety‐nine people living in the Nottinghamshire area died of ARLD between 2012 and 2017. Seventy‐eight percent (*n* = 627) of the cohort had ARLD recorded as their primary cause of death. The mean age at death was 56.6 years (SD ± 11.8), 65.7% (*n* = 525) were male and 94.9%, (*n* = 714) were of White ethnicity. Sixty‐six percent were residents of Nottingham City (*n* = 525) and 65.3% (*n* = 522) were from the two most deprived neighbourhoods of Nottinghamshire (Table 1). Eighty‐six percent (*n* = 605) were admitted as an emergency when first diagnosed with ARLD. The number of cases and crude rates by age group for men and women is provided in Figure 1.

**TABLE 1:** Characteristics of the cohorts

	Whole cohort	Early diagnosis^a^	Late diagnosis^b^	*P‐*value^c^
	*N* = 799	*N* = 400	*N* = 399	
Age at death	56.6 (±11.8)	56.6 (±11.8)	57 (±11.7)	0.28
*Sex*				
Male	65.7% (525)	65.8% (263)	65.7% (262)	0.987
*Ethnicity*				0.018
White	94.9% (714)	94.5% (364)	95.4% (350)	
Other	5.1% (38)	5.5% (21)	4.6% (17)	
Missing	47	15	32	
*Area of residence*				0.436
Nottingham city	65.7% (525)	64.8% (259)	66.7% (266)	
Nottinghamshire	34.3% (274)	35.2% (141)	33.3% (133)	
*Deprivation quintile*				0.975
Most deprived 1	37.4% (299)	37.8% (151)	37.1% (148)	
2	27.9% (223)	28.5% (114)	27.3% (109)	
3	17.9% (143)	16.8% (67)	19.0% (76)	
4	10.0% (80)	10.0% (40)	10.0% (40)	
Least deprived 5	6.8% (54)	7.0% (28)	6.5% (26)	
*Mode of alcohol‐related liver disease diagnosis*				<0.001
Routine/elective	13.5% (94)	18.0% (72)	5.5% (22)^d^	
Emergency	86.6% (605)	82.0% (328)	69.4% (277)^d^	
No record	100	0	100	
Median number of inpatient admissions	5 (3–10)	8 (5–14)	3 (2–5)	<0.001
Median number of outpatient appointments	16 (7–29)	23 (13–37)	9 (4–19)	<0.001
Median number of emergency attendances	4 (2–8)	6 (3–12)	3 (2–5)	<0.001
*Year of death*				0.518
2012	111 (13.9%)	61 (15.3%)	50 (12.5%)	
2013	130 (16.3%)	60 (15.0%)	70 (17.5%)	
2014	123 (27.9%)	57 (14.3%)	66 (16.5%)	
2015	134 (16.8%)	74 (18.5%)	60 (15.0%)	
2016	141 (17.6%)	71 (17.8%)	70 (17.5%)	
2017	160 (20.0%)	77 (19.3%)	83 (20.8%)	

*Note*: Mean (SD), Median (interquartile range), % (number).

^a^ Diagnosis made >6 months prior to date of death.

^b^ Diagnosis made <6 months prior to date of death.

^c^ The *P*‐value for significance of difference between early versus late diagnosis.

^d^ Excluding patients with no record.

### 3.2 Timing of liver diagnosis

Thirteen percent (*n* = 100) of patients who died from ARLD had no hospital episode (inpatient admission, A&E or OPD attendance) with a diagnosis code indicating ARLD in any year of HES activity. A further 37.4% (*n* = 299) had their first diagnosis of ARLD within 6 months of their death, totalling 399 (49.9%) patients in the late diagnosis cohort.

In the early diagnosis group (50.1%, *n* = 400), 40% had their first ARLD diagnosis more than 5 years prior to their death. The only significant difference between the two cohorts was that elective admission for the first ARLD diagnosis was recorded more frequently among those with an early diagnosis compared to those with a late diagnosis (18.0%, *n* = 72 vs 5.5%, *n* = 22, *P* < 0.001) (Table 1).

### 3.3 Missed opportunities for intervention

#### 3.3.1 Frequency and speciality of attendance

In the 5 years prior to their death from ARLD patients had a median of five hospital admissions (interquartile range 3–10), 4 A&E attendances (interquartile range 2–8) and 16 OPD attendances (interquartile range 7–29) (Table 1). Most inpatient admissions were via general medicine 88.7% (*n* = 709), gastroenterology 64.1% (*n* = 512) or hepatology 37.3% (*n* = 298). Attendance at OPD was coded to gastroenterology 54.2% (*n* = 433), general medicine 52.6% (*n* = 420), trauma and orthopaedics 35.7% (*n* = 285), hepatology 31.6% (*n* = 253) and diagnostic imaging 26.4% (*n* = 211) (Table 2).

**TABLE 2:** Top 10 inpatient and outpatient speciality interaction

	Whole cohort	Early diagnosis^a^	Late diagnosis^b^
*Inpatient speciality of care*			
General medicine	88.7% (709)	87.8% (351)	39.1% (117)
Gastroenterology	64.1% (512)	66.5% (266)	18.5% (55)
Hepatology	37.3% (298)	31.3% (125)	2.5% (7)
General surgery	25.4% (203)	26.8% (107)	12.8% (38)
Trauma and orthopaedics	17.1% (137)	19.0% (76)	12.0% (36)
Accident and emergency	15.0% (122)	19.0% (76)	5.05 (15)
Thoracic medicine	14.0% (108)	9.0% (36)	4.0% (12)
Geriatric medicine	11.0% (87)	11.3% (45)	2.8% (8)
Adult mental illness	10.0% (78)	12.0% (48)	6.8% (20)
Cardiology	7.0% (57)	7.3% (29)	2.8% (8)
*Outpatient speciality of care*			
Gastroenterology	54.2% (433	65.0% (260)	19.8% (59)
General medicine	52.6% (420)	62.4% (250)	25.5% (76)
Trauma and orthopaedics	35.7% (285)	38.3% (153)	28.1% (84)
Hepatology	31.6% (253)	39.3% (157)	10.5% (31)
Diagnostic imaging (from 2008 to 2009)	26.4% (211)	28.3% (113)	16.0% (48)
Ophthalmology	17.0% (139)	19.5% (78)	11.5% (34)
Cardiology	16.0% (124)	16.3% (65)	10.0% (30)
Adult mental illness	15.0% (119)	18.3% (73)	9.0% (27)
General surgery	13.0% (100)	15.0% (60)	8.0% (24)
Thoracic medicine	11.0% (91)		
Total	799	400	299^c^

*Note*: % (number).

^a^ Diagnosis made >6 months prior to the date of death.

^b^ Diagnosis made <6 months prior to the date of death.

^c^ Excluding patients with no record.

Those in the early diagnosis group had a significantly higher number of hospital admissions, A&E attendances and OPD appointments over the 5 years prior to death compared to late diagnosis (all *P* < 0.001, Table 1). The inpatient and OPD speciality activity for early and late diagnosis groups largely mirrored that of the whole cohort with the following exceptions: the late diagnosis group had fewer hepatology inpatient admissions and OPD attendances compared to the early diagnosis group (inpatient admission 2.5%, *n* = 7 vs 31.3%, *n* = 125), OPD attendance (10.5%, *n* = 31 vs 39.3%, *n* = 157, Table 2).

#### 3.3.2 Diagnoses related to attendance

Among the whole cohort, ARLD and mental and behavioural disorders due to alcohol were the top primary and secondary diagnoses. ARLD formed 63.4% (*n* = 507) of primary diagnoses and 78.5% (*n* = 628) of secondary diagnoses. Mental and behavioural disorders due to alcohol formed 25.7% (*n* = 206) of primary diagnoses and 77.1% (*n* = 616) of secondary diagnoses. Of those who received an early diagnosis, 59.5% (*n* = 238) were admitted due to ARLD in the 5 years before death. The second commonest primary diagnosis for the early diagnosis group and commonest for the late diagnosis group was mental and behavioural disorders due to the use of alcohol at 32.3% (*n* = 129) and 14.3% (*n* = 57), respectively (Table 3).

**TABLE 3:** Top 10 ICD‐10 primary and non‐primary (secondary) inpatient diagnoses

Primary ICD‐10 diagnosis		Non‐primary ICD‐10 diagnosis	
*Whole cohort*			
Alcoholic liver disease^a^	63.4% (507)	Alcoholic liver disease^a^	78.5% (628)
Mental and behavioural disorders due to alcohol^a^	25.7% (206)	Mental and behavioural disorders due to alcohol^a^	77.1% (616)
Other diseases of digestive system	21.0% (171)	Ascites	58.0% (465)
Oesophageal varices	14.0% (109)	Mental and behavioural disorders due to tobacco	55.0% (438)
Pneumonia, organism unspecified	13.0% (103)	Personal history of certain other diseases	54.0% (428)
Abdominal and pelvic pain	13.0% (103)	Other diseases of the liver	51.0% (410)
Ascites	11.0% (86)	Other disorders of fluid, electrolyte, and acid–base balance	50.0% (398)
Syncope and collapse	9.0% (75)	Acute renal failure	48.0% (383)
Peritonitis	9.0% (72)	Problems related to lifestyle	38.0% (302)
Hepatic failure, not otherwise specified	9.0% (71)	Depressive episode	32.0% (257)
Total	799		
*Early diagnosis*			
Alcoholic liver disease^a^	59.5% (238)	Alcoholic liver disease^a^	79.8% (319)
Mental and behavioural disorders due to use of alcohol^a^	32.3% (129)	Mental and behavioural disorders due to use of alcohol^a^	75.8% (303)
Other diseases of the digestive system	20.5% (82)	Personal history of other diseases	51.3% (205)
Oesophageal varices	17.8% (71)	Ascites	50.0% (200)
Abdominal and pelvic pain	14.8% (59)	Mental and behavioural disorders due to tobacco	49.0% (196)
Syncope and collapse	10.0% (40)	Other diseases of liver	47.0% (188)
Ascites	10.0% (40)	Problems related to lifestyle	41.0% (164)
Pain in throat and chest	9.8% (39)	Other disorders of fluid, electrolyte, and acid–base balance	33.8% (135)
Pneumonia	8.5% (34)	Essential (primary) hypertension	31.3% (125)
Other anaemias	7.8% (31)	Depressive episode	31.0% (124)
Total	400		
*Late diagnosis*			
Mental and behavioural disorders due to use of alcohol^a^	14.3% (57)	Mental and behavioural disorders due to use of alcohol^a^	32.8% (98)
Other diseases of the digestive system	5.5% (22)	Mental and behavioural disorders due to tobacco	26.6% (79)
Abdominal and pelvic pain	4.8% (19)	Personal history of other diseases	15.0% (45)
Syncope and collapse	4.3% (17)	Depressive episode	15.0% (45)
Pain in throat and chest	4.0% (16)	Essential (primary) hypertension	14.3% (43)
Open wound of head	2.8% (11)	Problems related to lifestyle	13.8% (41)
Oesophageal varices	2.5% (10)	Unspecified fall	9.0% (27)
Gastritis and duodenitis	2.5% (10)	Abnormal results of function studies	7.5% (22)
Acute pancreatitis	2.5% (10)	Other disorders of fluid, electrolyte, and acid–base balance	6.5% (19)
Fracture of femur	2.5% (10)	Other diseases of liver	6.5% (19)
Total	299^b^		

*Note*: % (number).

^a^ Wholly attributable alcohol conditions.

^b^ Excluding patients with no record. ICD‐10, International Classification of Diseases, 10th edition.

## 4 DISCUSSION

This large cohort of individuals with ARLD mortality had a median of 25 hospital attendances each in the preceding 5‐year period which represents significant missed opportunities for intervention. In addition, half of those dying with ARLD recorded a late diagnosis of ARLD. This is despite repeated health‐care interactions, including health issues directly associated with alcohol.

These findings build on a recent population‐based study for all‐cause cirrhosis exploring the impact of hospitalisation compared to ambulatory care on one and 5‐year survival [22]. In their study cohort, just over half (*n* = 2698, 52.7%) received the first diagnosis of liver disease in ambulatory care, whereas *n* = 2420 (47.3%) were as emergency hospital admissions. Emergency hospital admissions were further associated with a significant reduction in overall survival independent of the stage of cirrhosis [22]. In the present study, we observed that an emergency admission was significantly associated with a delay in diagnosis of ARLD. This could explain a higher risk of mortality in this group likely due to a missed opportunity for early diagnoses of ARLD and intervention. The age, gender and ethnic distribution are well‐matched with the UK national statistics for ARLD‐related mortality [23]. The existing alcohol paradox was again noted, where the most deprived had the highest mortality [24].

Alcohol misuse is a complex medical disorder with significant associated mental health disorders [25] and requires a holistic multidisciplinary approach for effective management. The dual diagnosis of alcohol and mental health is associated with the worst clinical and social outcomes and with significant stigma [26]. It is estimated that in the UK one‐third of people with mental health disorders have a coexisting substance use disorder [26, 27]. In the present study, over a quarter of patients had mental and behavioural disorders due to alcohol as a primary diagnosis and a further two‐thirds as a secondary diagnosis. Despite this high prevalence, the services to deal with dual diagnosis are often inadequate [26]. The chronic lack of coordination among front‐line health‐care professionals increases the vulnerability of these individuals to not getting the right treatment and missing an opportunity [27].

By highlighting where this cohort has encountered health services, we have helped to identify areas where the implementation of alcohol identification and brief advice (AIBA) could have the most impact. The use of AIBA has a proven role in reducing alcohol consumption and subsequent harm [28]. Over the last decade, there has been a drive in the UK to promote AIBA across a range of services [29, 30]. The National Institute of Clinical Excellence 2011 guidelines state that staff working in the NHS and involved in caring for people at risk of alcohol misuse should be competent in identifying harmful alcohol intake and delivering a brief intervention [31].

The individuals studied had a median attendance of 16 times at OPD in their final 5 years of life. The cohort attended OPD under a variety of specialities other than gastroenterology or hepatology, including trauma and orthopaedics, ophthalmology, ear, nose and throat and neurology. The message that health improvement is the responsibility of all health‐care professionals is not yet fully embedded, despite assurances to the contrary. The 2010 position statement from The Royal College of Surgeons of England, stressed the surgeon's role in capitalising on ‘teachable moments’ in the OPD by screening patients for alcohol misuse followed by a brief intervention [32]. In order to address alcohol misuse treatment services must be more accessible. As must education for health‐care professionals across the board on how to integrate early diagnosis of alcohol misuse and interventions into their practice [13]. There is a growing body of evidence supporting clinician lead integrated multidisciplinary care models to provide person‐centred care for alcohol misuse [33, 34]. A recent study has demonstrated that the incorporation of liver specialist nurses into general practice was feasible and increased the yield of the new diagnosis of liver disease [35].

### 4.1 Strengths and limitations

This study used a novel approach by linking hospital episode and mortality data for ARLD to identify common factors associated with a delay in diagnosis and missing potential opportunities for alcohol misuse interventions. Previous studies lack a focus on the patient level interaction with health‐care services in any setting where an opportunity for AIBA can arise. The use of validated ARLD codes with a high positive predictive value and sensitivity [36, 37] means we can be confident that these findings do reflect individuals with ARLD. Conversely, some individuals misclassified will have been missed.

A limitation of the study was the inability to link HES data with primary care or community services, as this would have enabled an evaluation of the community alcohol use disorder pathway and a view of whether referral to services was made following an ARLD diagnosis. This also risks missing patients who were diagnosed with ARLD in primary care, however, it is unlikely that a diagnosis of significant liver disease would not be associated with a resultant hepatology specialist assessment in secondary care. A recent study assessing primary and secondary care linked data to identify all‐cause services found significant but not perfect overlap between the two health‐care settings [22]. We are assuming that in the late diagnosis cohort hospital attendances indicate a missed opportunity, however, there is no data in our study to know if alcohol interventions were undertaken and unsuccessful for some patients.

During the proposed study period only patients admitted to Nottingham University Hospitals identified by nursing or medical staff as having alcohol problems were referred to alcohol liaison services [38]. Existing evidence suggests hospital staff are inconsistent in identifying alcohol misuse among hospitalised patients [39]. Those attending outpatients or in accident emergency (the majority of the cohort) would not have had this opportunity, limiting the use of brief interventions in the study population. To date, there is a lack of provision of AIBA in these clinical areas in secondary care both locally at Nottingham University Hospitals NHS Trust and nationally in the UK.

Since this study introduction of universal alcohol screening for in‐patients [12] has been implemented in this NHS Trust. However, such an initiative is not present throughout UK secondary care and represents a key opportunity to engage people in alcohol services. Although several types of brief advice exist, the ideal content to engage a positive impact is not yet clear. This emphasises the importance of studying the patient level impact of the different types of brief interventions to promote an integrated health‐care approach with an evidenced‐based choice suited to individual needs [28].

## 5 CONCLUSION

ARLD is a preventable cause of premature mortality. The relatively younger, male, and most deprived are at major risk. These individuals are frequent health‐care attendees and there are numerous opportunities to identify alcohol misuse and intervene. However, these opportunities are not being maximised. We have identified that OPD settings (including those unrelated to liver disease) are important targets as they represent the most common contacts with the health service—more so than A&E. It also indicates the need for further study of optimal pathways and interventions in accessing effective AIBA, referral pathways, diagnostics and treatment of alcohol misuse. There is also an urgent need to review local and national level alcohol policy to implement population‐based interventions, such as minimum unit pricing, alcohol access and alcohol labelling which have proven benefit in reducing alcohol‐related harm.

## AUTHOR CONTRIBUTIONS

Rebecca Elleray contributed to study proposal, ethical approval, data collection and analysis and manuscript review. Mohsan Subhani contributed to data analysis and interpretation, report writing and manuscript review. Joanne R. Morling contributed to data interpretation and review of the manuscript. Jane Bethea contributed to developing study idea, supervision of the study process and review of the manuscript. Stephen Ryder contributed to developing the study idea, supervision of study process and review of the manuscript.

## FUNDING

JRM (co‐author) receives salary support from a Medical Research Council Clinician Scientist Fellowship (grant number MR/P008348/1).

## CONFLICT OF INTEREST

The authors declare no conflict of interest.

## Supporting information


**Table S1.** ICD‐10 code for wholly attributable alcohol conditions included the study.
**Table S2.** Multivariable association between late diagnosis and patient characteristics.
