# Supplementary material for: Alcohol‐related liver disease mortality and missed opportunities in secondary care: A United Kingdom retrospective observational study
Source: Drug Alcohol Rev. 2022 May 31;41(6):1331–40. doi: 10.1111/dar.13482 (PMC9541852; doi:10.1111/dar.13482)
Supplement: Supplementary file 1 — Table S1. ICD‐10 code for wholly attributable alcohol conditions included the study. Table S2. Multivariable association between late diagnosis and patient characteristics. [file DAR-41-1331-s001.docx]

**Table S1**. ICD-10 code for wholly attributable alcohol conditions included the study

| **Conditions** | **ICD-10 code^a^** |
| --- | --- |
| *Mental and behavioural disorders due to use of alcohol (F10.X)* |  |
| Alcohol intoxication | F10.0 |
| Harmful use of alcohol | F10.1 |
| Alcohol dependence | F10.2 |
| Alcohol withdrawal state | F10.3 |
| Alcohol withdrawal state with delirium | F10.4 |
| Alcohol induced psychotic disorders | F10.5 |
| Alcohol induced amnestic disorders | F10.6 |
| Alcohol induced residual and late onset psychotic disorders | F10.7 |
| *Liver disorders due to alcohol (K70.x)* |  |
| Alcoholic fatty liver | K70.0 |
| Alcoholic hepatitis | K70.1 |
| Alcoholic fibrosis and sclerosis of liver | K70.2 |
| Alcoholic cirrhosis of liver | K70.3 |
| Alcoholic hepatic failure | K70.4 |
| Alcoholic liver disease, unspecified | K70.9 |

ICD-10, International Classification of Disease 10^th^ edition.

**Table S2.** Multivariable association between late diagnosis and patient characteristics

|  | **Unadjusted** | **Adjusted** | |
| --- | --- | --- | --- |
|  | Odds ratio (95% CI) | Odds ratio (95% CI) | *P* |
| Age at death | 1.01 (0.99-1.02) | 1.01 (0.99-1.02) | 0.2801 |
| *Sex* |  |  |  |
| Males | 1 |  | 0.987 |
| Females | 1.00 (0.75-1.34) | 1.00 (0.74-1.35) |  |
| *Ethnicity* |  |  |  |
| White | 1 |  | 0.0179 |
| Non-White | 0.84 (0.44-1.62) | 0.78 (0.39-1.55) |  |
| *Area of residence* |  |  |  |
| Nottinghamshire | 1 |  | 0.4356 |
| Nottingham city | 0.92 (0.68-1.23) | 1.16 (0.80-1.70) |  |
| *Deprivation quintile* |  |  |  |
| Most deprived 1 | 1 |  | 0.9747 |
| 2 | 0.98 (0.69-1.38) | 1.02 (0.70-1.47) |  |
| 3 | 1.16 (0.78-1.72) | 1.13 (0.73-1.74) |  |
| 4 | 1.02 (0.62-1.67) | 1.15 (0.67-1.96) |  |
| Least deprived 5 | 0.95 (0.53-1.69) | 1.02 (0.54-1.90) |  |
| *Mode of ARLD diagnosis^a^* |  |  |  |
| Routine/elective | 1 | 1 | <0.001 |
| Emergency | 2.76 (1.67-4.57) | 2.53 (1.50-4.26) |  |
| *Year of death* |  |  |  |
| 2012 | 1 |  | 0.5177 |
| 2013 | 1.42 (0.86-2.37) | 1.46 (0.86-2.45) |  |
| 2014 | 1.41 (0.84-2.36) | 1.51 (0.89-2.56) |  |
| 2015 | 0.99 (0.60-1.64) | 1.04 (0.62-1.76) |  |
| 2016 | 1.2 (0.73-1.98) | 1.19 (0.71-1.99) |  |
| 2017 | 1.32 (0.81-2.14) | 1.29 (0.78-2.13) |  |

^a^At the time when first diagnosed with ARLD. ARLD, alcohol-related liver disease; CI, confidence interval.
